# An exploration of the complex biogeographical history of the Neotropical banner-wing damselflies (Odonata: Polythoridae)

**DOI:** 10.1186/s12862-020-01638-z

**Published:** 2020-06-24

**Authors:** Melissa Sánchez-Herrera, Christopher D. Beatty, Renato Nunes, Camilo Salazar, Jessica L. Ware

**Affiliations:** 1 Department of Biology, Faculty of Natural Sciences, Universidad del Rosario, Bogota, DC Colombia; 2 Federated Department of Biological Sciences. Rutgers, The State University of New Jersey, Newark, NJ USA; 3 Department of Ecology & Evolutionary Biology, Cornell University, Ithaca, NY USA; 4 Departament of Biology, The City University of New York, New York, NY USA; 5 American Museum of Natural History, New York, NY USA

**Keywords:** Damselfly, Neotropical region, Zygoptera, Ancestral areas, Andean uplift, Marine incursions, Central America seaway, Odonata

## Abstract

**Background:**

The New World Tropics has experienced a dynamic landscape across evolutionary history and harbors a high diversity of flora and fauna. While there are some studies addressing diversification in Neotropical vertebrates and plants, there is still a lack of knowledge in arthropods. Here we examine temporal and spatial diversification patterns in the damselfly family Polythoridae, which comprises seven genera with a total of 58 species distributed across much of Central and South America.

**Results:**

Our time-calibrated phylogeny for 48 species suggests that this family radiated during the late Eocene (~ 33 Ma), diversifying during the Miocene. As with other neotropical groups, the Most Recent Common Ancestor (MRCA) of most of the Polythoridae genera has a primary origin in the Northern Andes though the MRCA of at least one genus may have appeared in the Amazon Basin. Our molecular clock suggests correlations with some major geographical events, and our biogeographical modeling (with BioGeoBEARS and RASP) found a significant influence of the formation of the Pebas and Acre systems on the early diversification of these damselflies, though evidence for the influence of the rise of the different Andean ranges was mixed. Diversification rates have been uniform in all genera except one—*Polythore*—where a significant increase in the late Pliocene (~ 3 mya) may have been influenced by recent Andean uplift.

**Conclusion:**

The biogeographical models implemented here suggest that the Pebas and Acre Systems were significant geological events associated with the diversification of this damselfly family; while diversification in the tree shows some correlation with mountain building events, it is possible that other abiotic and biotic changes during our study period have influenced diversification as well. The high diversification rate observed in *Polythore* could be explained by the late uplift of the Northern Andes. However, it is possible that other intrinsic factors like sexual and natural selection acting on color patterns could be involved in the diversification of this genus.

## Background

The New World tropics is a region of amazingly high diversity in a variety of plants and animals, and has a complex history [1]. Shifting continents, multiple instances of mountain building, rivers that change their course, and the expansion and retreat of both freshwater and marine habitats all come together to make a complicated and dynamic foundation on which biological diversity has developed over the last 50 million years (Ma). These include the uplift of the different regions of the Andes, as well as the Venezuelan Highlands, the formation of the extensive Amazonian flood basin in the Miocene (Pre-Pebas, Pebas and Acre), the further development of the Amazon and Orinoco drainages, the dry/wet climate cycles of the Pliocene/Pleistocene, and the formation of the Panamanian Land Bridge between the Tumbes-Chocó-Magdalena regions and Central America [2–4].

In a number of groups (tropical frogs [5, 6], butterflies [7, 8], lupines [9], birds [10]) this changing geography has driven substantial—sometimes rapid—diversification, but not always in the same way. The diversification driven by these geographic changes often promotes niche diversification, with species taking advantage of the distinct ecological niches found in each local environment. This leads to adaptive speciation [11], further increasing diversity beyond the form of the landscape. Here we investigate the origins of diversity in the banner-winged damselflies (Odonata: Zygoptera: Polythoridae), a group that is distributed across much of Central and South America.

Polythoridae comprises 58 species across seven genera (*Chalcopteryx* Selys, *Chalcothore* De Marmels, *Cora* Selys, *Euthore* Selys, *Miocora* Calvert, *Polythore* Calvert and *Stenocora* Kennedy) that differ markedly in their distribution: some are found widely across the continent, while others are limited to a single region. While damselflies as a group are generalist predators in both their larval and adult forms, the types of aquatic habitats in which their larvae are found vary, with some species using lakes and ponds, while others inhabit streams and rivers. The larvae of Polythoridae prefer moving water; most species are found in small, fast-flowing streams in mountain regions, though some additionally exist within slow streams in the Amazon Basin [12]. These species display a diverse range of colors and patterns on both the wing and body within and among genera; previous work by Sánchez Herrera et al. [13, 14] has shown that for some genera, wing colors may be polymorphic, raising the question of what factors drive this color diversity.

We tested hypotheses concerning the diversity within Polythoridae, investigating the influence of geological events in shaping the distribution of the members of this family. Using relaxed-clock molecular methods we estimate divergence times within and among the genera of this family. We also investigate their biogeographical patterns of diversification in relation to the mountain building events of the Andes, as well as the extent of marine and freshwater environment incursions in different periods that might have influenced distribution and speciation in these genera. Specifically, we made the following predictions: (1) *We predict that the Andes uplift was a major driver of speciation in the family Polythoridae. Therefore, we also predict (2) that there were multiple interchanges between Neotropical regions, particularly from the Andean regions to the other biogeographical regions of Central and South America, for the different genera within* Polythoridae. Finally (3) *we predict that the family Polythoridae (or genera within) has experienced higher rates of diversification through recent evolutionary time*. Overall, this work represents a continental-scale biogeographical analysis of a neotropical family of damselflies. With their reliance on aquatic habitats, damselflies are likely to respond very differently to the landscape than plants, terrestrial vertebrates or insects; our analyses allow us to consider the formation of diversity in the modern Neotropics through an unexplored arthropod perspective.

## Results

### Phylogeny and time divergence estimation

**Fig. 1:**
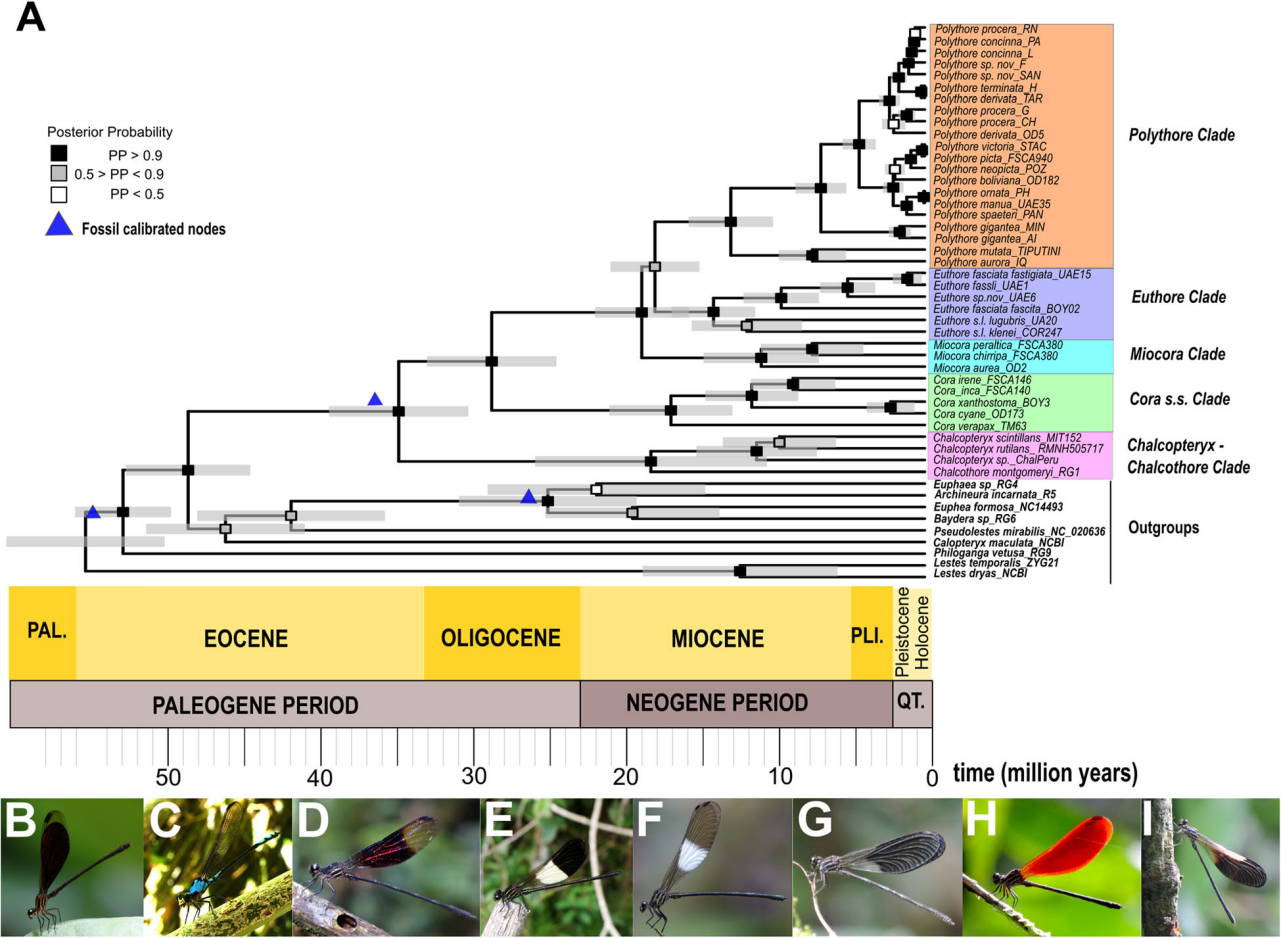
Phylogenetic relationships and representative pictures of all the main clades of the family Polythoridae. **a** Bayesian time-calibrated best tree obtained in BEAST v1.8.4 with median node ages, 95% High Probability Density (HPD) intervals for each node age displayed in grey bars, and nodes posterior probability support (PP) represented with squares at each node (see legend top-right). Fossil calibrated nodes are represented as blue triangles. Pictures at the bottom are from a representative species of the five major clades **b**. *Chalcopteryx rutilans*, *Chalcothore – Chalcopteryx* clade©Adolfo Cordero. *C. Cora xanthostoma*, *Cora* s.s*.* clade© Cornelio Bota. D. *Miocora aurea, Miocora* Clade©Cornelio Bota. E*. Euthore fasciata fasciata*, *Euthore* s.l clade© Cornelio Bota. The *Polythore* clade which has a high diversity on wing coloration is represented by: F*. Polythore mutata*, Amazon clade© Adolfo Cordero; G. *Polythore gigantea*, West Andes clade© Melissa Sanchez-Herrera, H. *Polythore concinna*, Northeastern Andes clade© Melissa Sanchez-Herrera and I. *Polythore ornata*, Southeastern Andes clade© Melissa Sanchez-Herrera

Our IQTree ML phylogenetic reconstruction, as well as our BEAST time calibrated tree, were completely concordant with the clades previously recovered by Sánchez Herrera et al. [13] (Fig. 1, Fig. S1), though our current dataset includes 12 additional species. The family Polythoridae separated from our Outgroups ~ 46 Ma (49–42 Ma 95% HPD [High Probability Density intervals], pp. [posterior probability] = 0.9125). Our time divergence reconstruction suggests that the MRCA (Most Recent Common Ancestor) of the family Polythoridae diverged ~ 33 Ma (37–29 Ma 95% HPD, pp. = 1) during the late Oligocene and early Miocene boundary. The *Chalcopteryx –Chalcothore* crown clade itself began to diversify later during the early Miocene ~ 17.13 Ma (24.17–9.6 Ma 95% HPD, pp. = 1), while the crown group of all of the other Polythoridae genera (*Cora s.s., Miocora, Euthore* and *Polythore* clades) began to diversify ~ 27 Ma (23.10–31.20 Ma 95% HPD, pp. = 1) during the late Oligocene. *Chalcothore* separated from *Chalcopteryx* ~ 17.13 Ma (24.17–9.6 Ma 95% HPD, pp. = 1) around the mid Miocene, however the crown group of *Chalcopteryx* only diversified during the late Miocene ~ 10.5 Ma (13.8–6.24 Ma 95% HPD, pp. = 0.8). The MRCA of the *Cora s.s* clade separates from all the other taxa ~ 27 Ma, however its crown group of *Cora s.s.* appears ~ 15.8 Ma (19.8–12.13 Ma 95% HPD, pp. = 1) around the end of the early Miocene (Fig. 1). The crown group of the *Miocora* clade appears ~ 10.2 Ma (13.9–6.62 Ma 95% HPD, pp. = 1), a few million years after *Cora s.s* during the mid-Miocene*,* although the MRCA of *Miocora* (separate from the *Euthore* and *Polythore* clade) appears ~ 17.7 Ma (20.7–14.8 Ma 95% HPD, pp. = 1) during the early Miocene epochs (Fig. 1). The two sister clades of *Euthore* and *Polythore* diverged from each other around the same epoch as *Miocora,* ~ 16.9 Ma (19.4–13.8 Ma 95% HPD, pp. = 0.65), but both form crown groups concurrently around the mid Miocene, though *Euthore* estimates are slightly older than those for *Polythore* (Fig. 1). *Euthore* crown group diversified ~ 13.2 Ma (15.8–10.6 Ma 95% HPD, pp. = 1) while *Polythore* seems to be ~ 12.1 Ma (14.8–9.55 Ma 95% HPD, pp. = 1). Within the *Euthore* clade we recovered two sister clades: *Euthore* sensu stricto (e.i. *E. fasciata fasciata, E. fasciata fastigiata, E. fassli, E. sp. nov),* and what used to be part of *Cora* (i.e. *E. lugubris* and *E. klenei).* Within the *Euthore* clade the divergence times recovered suggest that *Euthore* sensu stricto*, ~* 8.9 Ma (11.3–6.6 Ma, 95% HPD; pp. = 0.8), is younger that the previous classified *Cora* species within this clade, ~ 11.13 Ma (13.45–6.6 Ma, 95% HPD; pp. = 0.7).

In the highly speciose *Polythore clade* we recovered the divergence times for all the previous geographical clades reported in Sanchez Herrera et al [13] (Fig. 1, Fig. S1). The crown group of the Amazonian clade appeared ~ 7.03 Ma (7.33–3.04 Ma 95% HPD, pp. = 1), while the Andean clade was ~ 6.5 Ma (6.67–2.89 Ma 95% HPD, pp. = 1). Within the Andean clade, the crown groups of the West and East Andean clades appeared ~ 1.56 Ma (6.55–3.42 Ma 95% HPD, pp. = 1) and ~ 4.1 Ma (7.93–3.5 Ma 95% HPD, pp. = 1), respectively. Moreover, estimates for the North (~ 2.28 Ma, 2.8–0.99 Ma 95% HPD, pp. = 1) and South Eastern (~ 1.96 Ma, 3.17–1.19 Ma 95% HPD, pp. = 1) crown groups suggest they appeared around the Pliocene - Pleistocene epochs (Fig. 1).

### Biogeographical reconstruction

**Fig. 2:**
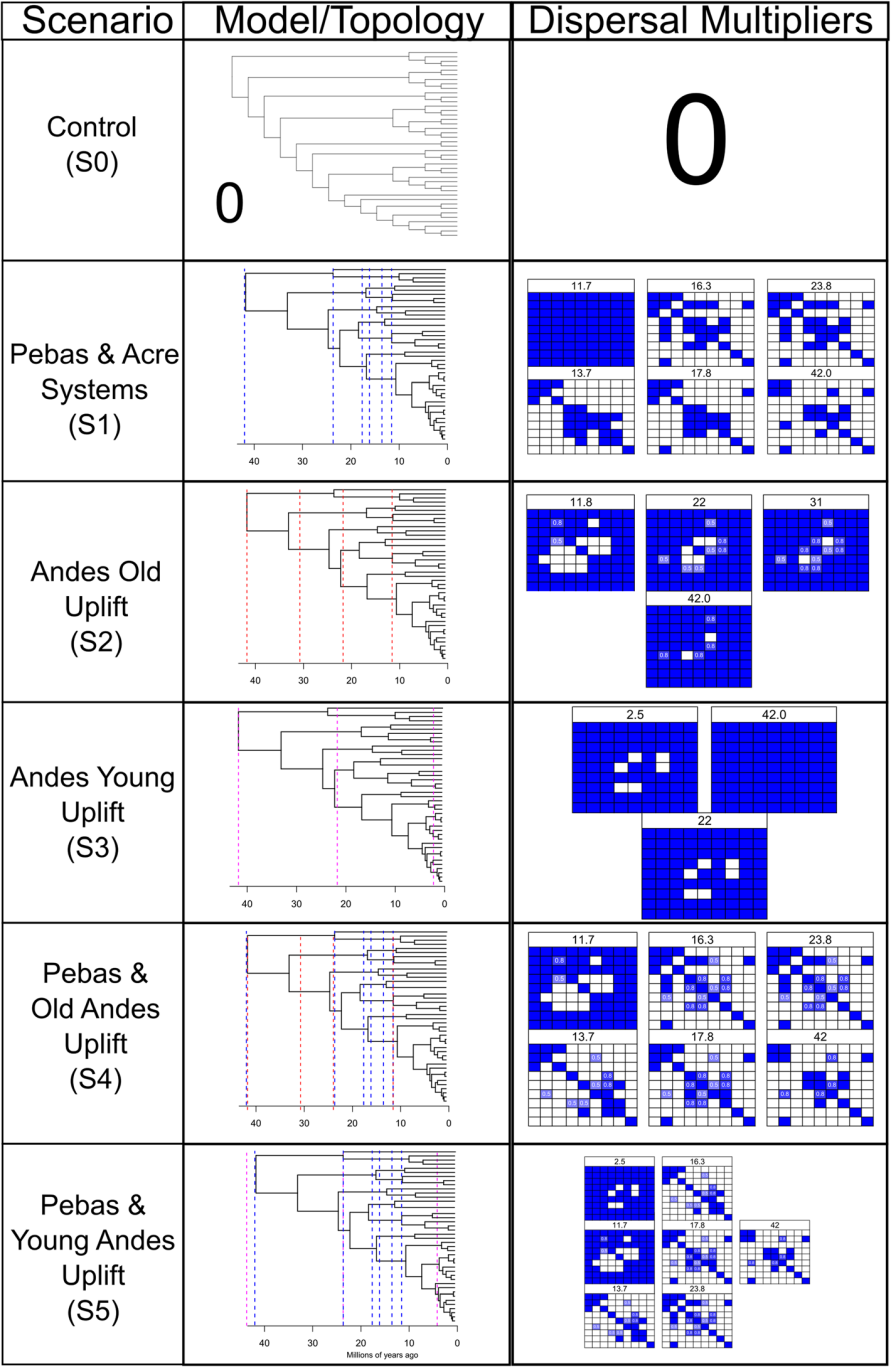
Designed Biogeographical Scenarios for the Ancestral Areas Reconstructions. All the five scenarios (S0-S5) tested in our biogeographical analyses using BioGeoBEARS accounting for the major geological events in South America. For each scenario, we show the tree topology model, time periods (i.e Millions of Years Ago) and dispersal multiplier matrices across all tested nine areas. The blue (1) to white (0) gradient represents the probability of dispersion across the areas. The Pebas and Acre Systems scenarios were designed using a combination of Hoorn et al. 2010 and Jaramillo et al. 2017, while for all Andes scenarios we used a combination of Hoorn et al. 2010 supplementary material and Gregory-Wodzicki 2000

**Fig. 3:**
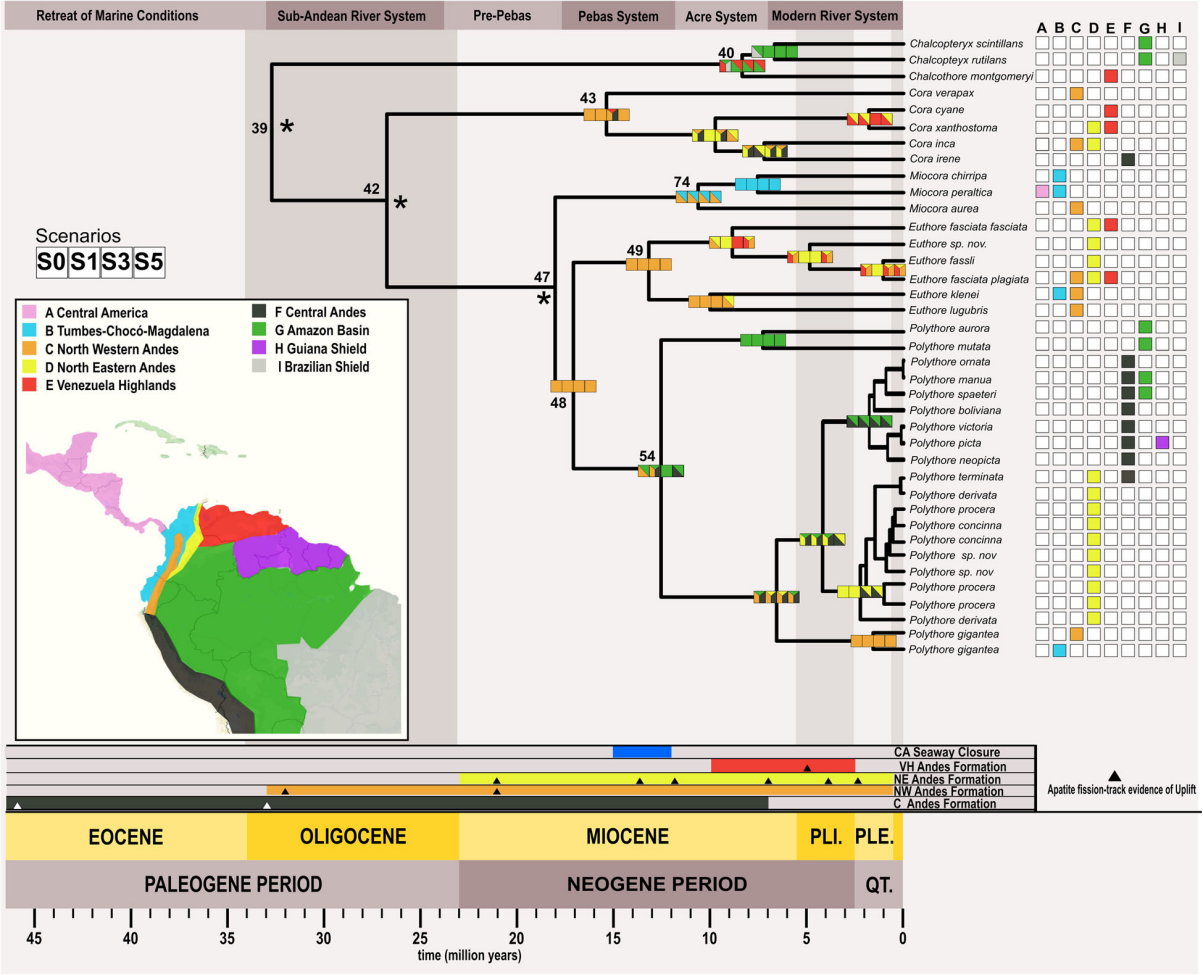
Time-Calibrated phylogenetic tree showing the Most Likely Ancestral Areas (MLAA) reconstructions for the best fitted tested scenarios. At the bottom-left there is a color-coded map showing the nine tested biogeographical regions used within the analyses. To the right of all the tips of the tree there is a color-coded matrix representing the current distribution of all the taxa. All internal nodes display four squares representing the most likely areas for the S0, S1, S3 and S5 scenarios at each node. Above, there is a banner showing the major marine incursion events and below there are banners representing the time ranges of the following geological events: formation of the Central America Seaway Closure (Blue), the Central Andes (Grey), the Northwest Andes (Orange), the Northeast Andes (Yellow) and Venezuelan Highlands (Red). The small black triangles represent the apatite track evidence of uplift extracted from Hoorn et al. 2010

We designed and tested five different scenarios—Control (S0), Pebas & Acre Systems (P&AS, S1), ANDES Old uplift (ANDES O, S2), ANDES young uplift (Andes Y, S3), Pebas & Old Andes (P&OA, S4) and Pebas & Young Andes (P&YA, S5)—to reconstruct the Ancestral Areas through multiple biogeographical models (e.g. DIVA, DEC or BayAreaLike) for the Polythoridae. The designated areas include; (A) Central America, (B) Tumbes-Choco-Magdalena, (C) North Western Andes, (D) North Eastern Andes, (E) Venezuela Highlands, (F) Central Andes, (G) Amazon Basin, (H) Guiana Shield, and (I) Brazilian Shield. For the control scenario, we tested if the phylogenetic relationships (i.e. cladogram) alone will recover the Ancestral Areas without imposing any restrictions (e.g. dispersal and adjacency) of the geological events including the Andes uplift and Marine incursions of South America (see details in Fig. 2). We selected the best biogeographical models for all scenarios (S0, S1, S2, etc.) using the AICc criteria from BioGeoBEARS. The lowest AICc value for all the scenarios tested was DEC or DEC + J (Table S2). However, following the Ree and Sanmartín [15] critique of the DEC + J model, we reconstructed the DEC and next best fitted biogeographic model based on the AICc for each scenario (Table 2). For all the scenarios (S0 – S5) we reconstructed the ancestral areas using the best model selected—DEC and S-DEC—using BioGeoBEARS and RASP software, respectively. However, for the second best model for both the Control (S0) and the P&AS model (S1), we reconstructed the areas using the DIVALIKE and S-DIVA models, while for all the other scenarios (S2, S3, S4 and S5), the BayAreaLike model was reconstructed in BioGeoBEARS. The lowest AICc values across all the scenarios were for the control S0, which was followed very closely by the P&AS (S1) (Table 1). The next lowest AICc values were the P&YA (S5) and the Young ANDES (S3), while the others (S2 and S4) seem not to fit the diversification model. However, there were no major differences in the reconstructed ancestral areas (Table S2) when comparing among all five models in both BioGeoBEARS and RASP. To simplify, here we will describe the most likely ancestral areas estimated for the four scenarios with the lowest AICc values (Fig. 3). All reconstructions became problematic at the deeper nodes, so the likelihood values supporting the areas are lower than 70% in nodes 39, 42 and 47 (Fig. 3), suggesting uncertainty in the ancestral areas for the MRCA for the family Polythoridae (node 39), for the clade that includes *Cora* s.s, *Miocora, Euthore* and *Polythore* (node 42), and also for the MRCA of the *Miocora, Euthore* and *Polythore* clades (node 47). For the MRCA (node 48) of *Euthore* and *Polythore,* the Northwest Andes (C) is the most commonly predicted ancestral area for all scenarios (Fig. 3)*.* The MRCA (node 49) for the crown group of *Euthore* is consistently found in the Northwest Andes (C) in all scenarios (Fig. 3). The MRCA (node 43) for the crown group of *Cora* s.s. has an ancestral area in the Northwest Andes (C), with the exception of S3, where in addition the Central Andes (F) and Venezuelan highlands (E) are supported (Fig. 3). The MRCA (node 74) for the *Miocora* crown group is found in the Tumbes-Chocó-Magdalena Valley (B) and the Northwest Andes (C). The youngest MRCA (node 40) of the *Chalcopteryx-Chalcothore* crown group showed as more plausible areas the Amazon Basin (G), and the Venezuelan Highlands (E); S1 also supports the Brazilian Shield (I) (Fig. 3). Finally, the MRCA (node 54) of the *Polythore* crown group is the only group that shows different ancestral areas depending on the scenarios: for S0 the Northwest Andes (C) and the Amazon Basin (G), for S1 the Northwest Andes (C), the Amazon Basin (G) and the Central Andes (F), for S3 only the Amazon Basin (G), and for S5 the Central Andes (F) and the Amazon basin (G) (Fig. 3, Table S2). It is interesting to note that the different scenarios (S0-S5) within our BioGeoBEARS and RASP analyses did not produce fundamentally different ancestral areas; inclusion of the formation of the Pebas and Acre wetlands systems, as well as mountain building events, did not alter our results in comparison with a simple control scenario that allowed free dispersal between adjoining regions at different periods of geological time (Fig. 2). Our best selected S-DEC model implemented in RASP for the Pebas and Acre Systems (S1) scenario suggests several dispersal (27), and vicariance (9) events within the different genera of Polythoridae (Fig. S3); many of these involve movement within and between the different ranges of the Andes, as well as movement into the Amazon, Guiana Shield, Venezuela Highlands, the Tumbes-Chocó-Magdalena Valley, and Central America.

**Table 1:**
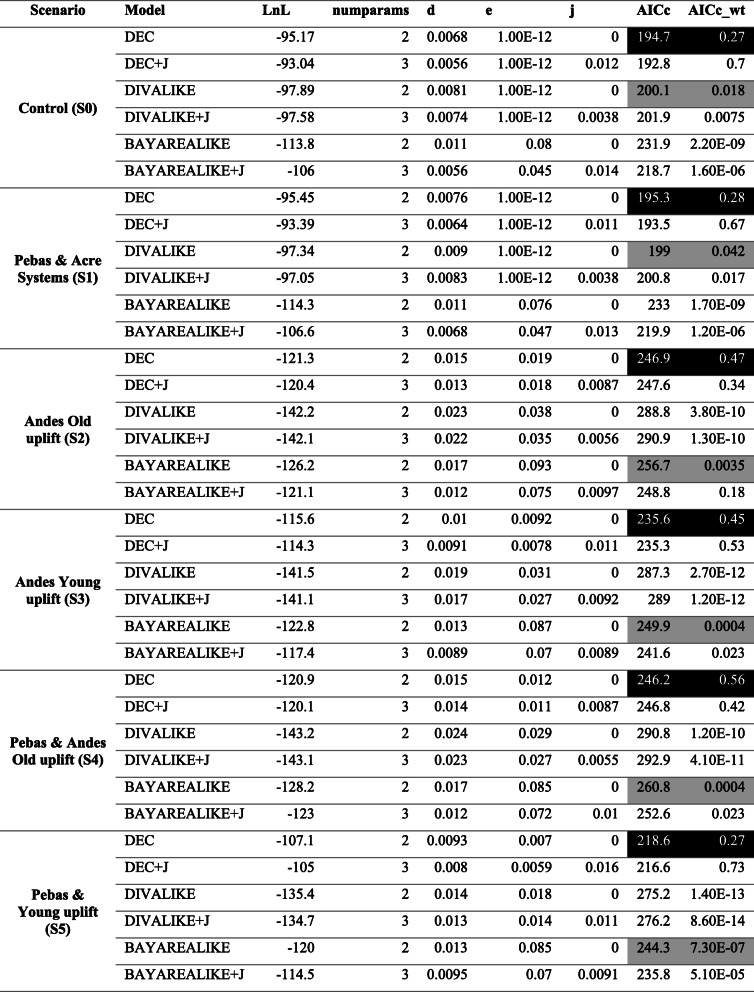
BioGeoBEARS model selection AICc values for each of the scenarios tested. The lowest AICc values are highlighted in black, and the second lowest in light grey

### Diversification analyses

**Fig. 4:**
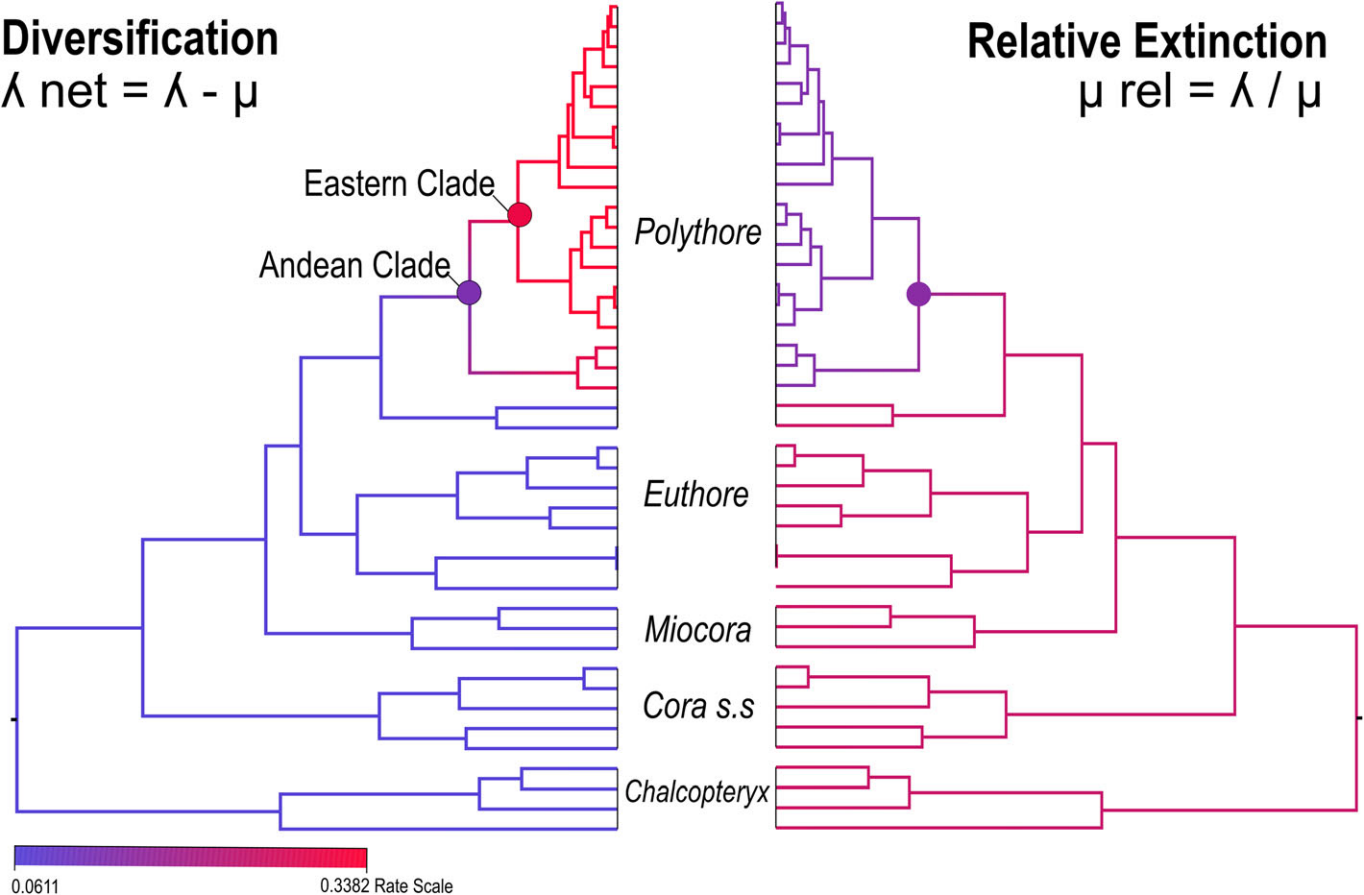
Diversification and Extinction rate shifts within family Polythoridae obtained using the RevBayes Branch-Specific diversification model. To the left, the net diversification rate is represented, while to the right is the relative extinction rate

To investigate the patterns of diversification and extinction rate variation through time and across lineages, we chose RevBayes [16, 17] to test the best diversification models and rate shifts, due to the high uncertainty associated with phylogenetic tree estimation. The Episodic Birth-Death with multiple time periods was the best model explaining the diversification pattern in this group of damselflies (Table S4). The estimated net diversification (λ-μ) and speciation (λ) rates show an increase, however the relative extinction (λ/μ) and extinction (μ) rates pattern seem to behave constantly through time (see Fig. S5). The Branch specific model detected a shift in diversification (λ-μ) rate corresponding to the Andean Clade in *Polythore* with the highest shift in the Eastern Clade (Fig. 4). When we observed the relative extinction (λ/μ) rate across the tree, we observed that this rate also decreases for this clade (Fig. 4). Our estimates show that the other clades within this family have a more constant diversification (λ-μ) and relative extinction (λ/μ) rates across the branches of the estimated tree.

## Discussion

Overall, our phylogenetic reconstruction was consistent with that previously reported by Sanchez-Herrera [13], despite the 12 additional taxa; the same major clades were recovered with high supported values (Fig. 1, Fig. S1). Our time divergence results suggest that the Polythoridae MRCA first appeared around the Eocene – Oligocene boundary, however most of the diversification within the family appeared during the mid-Miocene, Pliocene and Pleistocene epochs (Fig. 1). During these periods there were a few major geographical events occurring, including the uplift of different regions of the Andes, as well as the formation of the Pebas and Acre Systems [1, 18]. While the ancestral area for the MRCA of Polythoridae is uncertain, our biogeographical analyses suggest an origin—or at least early diversification—in the Northwestern Andes for most genera in Polythoridae, based on the ancestral areas of the different clades. *Chalcopteryx-Chalcothore* and *Polythore* are the only genera with a MRCA that may have had an Amazonian origin (Fig. 3, Table S2).

Our prediction that the uplift of the different regions of the Andes might be the driver of the diversification within Polythoridae was not completely supported by our results. Our biogeographical models found that the MRCA of most genera within Polythoridae seem to be better explained by the S1 model for the Pebas and Acre Systems (Table 1, Fig. 3). However, the Pebas and Acre Systems with the Young Andes uplift and the Old Andes uplift by itself (S5, S3) had a better fit (lower AICc) than the early uplift events of the Central Andes models (S2, S4). The latter may suggest that this event might be important to late diversification within the genera of this family. For example, the genus *Polythore* contains an Andean clade which shows a concordant pattern with the North Andes uplift. Likewise, *Cora* s.s, also shows a similar pattern separating two clades from the Northwest and Northeast Andes (Fig. 3) [11]. However, in terms of ancestral areas we found no significant differences across all the models we tested. Moreover, our results suggest that most of the diversification within Polythoridae is due to dispersal events rather than vicariance (Fig. S3). The distribution of those vicariance events (Fig. S3) in the time calibrated phylogeny is mainly within groups distributed along the Andes or that have representatives in Central America, reflecting the influence of these major geographical events on diversification.

What factors might be driving these biogeographical results? In particular for the family Polythoridae, the marine incursions and flooding events during the Miocene [19] likely had an effect on the distribution at the generic level. The Andes Cordillera seems to promote dispersal within the genera (Fig. 3, Fig. S3), and most likely these geological events have shaped the distribution of these damselflies, despite the mixed support of our models. These events may have cause significant changes in the biotic and abiotic conditions of different regions that may have induced Polythoridae damselfly dispersion to new suitable habitats. A previous assessment of Neotropical diversification for glass frogs (Allocentroleniae), an aquatic vertebrate, showed coincidental divergence time estimates with early uplifts of the Andes and lowland flooding events, similar to our results [6]. Moreover, our results suggest the Andes as a source of dispersal that did not play a role as a driver of diversification of lowland glass frog species [6]. We cannot deny that changes in the climatic conditions over that period of geological time may have had a strong influence on the biogeography of other organisms, including plants and herbivorous insects [20–22]. Our current analyses, which focused only on geological events, will not capture this complexity. Even though damselflies are generalist predators in both terrestrial and aquatic ecosystems, their distribution patterns might be affected by the vegetation and climatic barriers over evolutionary time.

A recent definition of the Neotropical forest relies on the combination of the following abiotic and biotic parameters: climate, floristic composition, vegetation structure and plant physiognomy [23]. Jaramillo [18] suggests that the formation of Neotropical terrestrial communities can be divided in two major phases, Cretaceous and Cenozoic, based on the defining parameters of Neotropical forests in these two geological eras. In our case the Cenozoic phase is more relevant, and it was characterized by the dominance of the flowering plants [24–27]. During the Neogene Period (i.e. Miocene and Pleistocene epochs), new biomes within the Neotropical regions flourished, such as savannas, dry forest, xerophytic forest, deserts, montane forest and paramos [18]. These biomes are determined by precipitation regimes rather than temperature [23]. However, questions regarding how climate and vegetation can influence the diversification of these damselflies remains untested.

We found multiple interchanges among the Amazon and Andean regions; many of them involved movement within and between the different ranges of the Andes, as well as movement into the Amazon, Guiana Shield, Venezuela Highlands, the Tumbes-Chocó-Magdalena Valley, and Central America (Fig. 3, Fig. S3). The different genera within Polythoridae showed an array of biogeographical patterns. The genus *Polythore* shows a pattern of dispersal from the Andean regions to the Amazon in some clades, as well as movement from the Northern to Central Andes (Fig. 3). On the other hand, *Euthore* moved from the Northwestern to Northeastern Andes and then to the Venezuelan highlands (Fig. 3). This genus is currently found at locations from 1000 to 2000 m in elevation; it is plausible that they colonized new habitats that became available during mountain building events in the Andes and Venezuelan highlands. *Cora* s.s and *Miocora* diversification was more likely driven by dispersal, as they cover a range of landscape types, and have moved across the Isthmus of Panama, the only groups in Polythoridae to do so. Interestingly, the age at which both of these genera diversified matches the age suggested by recent geological studies of the Central American Seaway closure during the middle of the Miocene [2, 28]. This closure may also influence the ITCZ (Inter Tropical Convergence Zone) [29] that will have affected precipitation regimes in the Neotropical Region [30].

These damselflies are restricted to fast flowing forested streams and waterfalls in montane forests; these habitat requirements are likely a limiting factor to their distribution [31–33]. However, as general predators they are not limited by the distribution of food sources such as the host plant for phytophagous insects (e.g. *Lepidoptera*). The potential for dispersal in the immature stage is limited to drift, moving with the flow of creeks; dispersal by adults through flight offers a potential for greater movement but is still likely to be in proximity to the stream or creek. Distribution between watersheds is thus likely to be limited, which could explain the vicariance patterns observed in our results.

Our analyses suggest that Polythoridae has been diversifying through an episodic pattern (Table S4). Speciation (λ) and net diversification (λ-μ) show an overall increase through time, while relative extinction (λ/μ) and extinction (μ) rates seem to remain somewhat constant through their evolutionary history. However, our branch-specific model of diversification shows a significant shift for the Andean Clade within the genus *Polythore* which might suggest that at least for this genus the intensified Andean uplift during the Pliocene has been promoting speciation (Fig. 3). The Andean uplift produced great modifications to the landscape; one of the major changes was in the flux of the hydrographic system towards the east, producing the actual Amazon and Orinoco basins. Furthermore, when the Andes reached their modern elevation by the end of the Miocene (i.e. 5–6 Ma) [1, 34–37], it generated two new biomes: cloud (montane) forest and paramo [18, 38]. The montane forest is the key habitat for the genus *Polythore,* and it has been suggested that the slopes of the Andes are an engine for speciation as the increase in topographic complexity generates diversity of microenvironments [1]. While all these species have relatively similar habitat requirements, they have generally disjunct distributions within the Andes, such that any local stream normally hosts only a single *Polythore* species, sometimes two species. The wing color diversity characteristic of *Polythore* is the highest within the Polythoridae--the central Andean *Polythore* have wings that include bands of black, orange and yellow patterns, while those of the Northeastern Andes have intense black and white patterning (except for *P. concinna*, which is orange). This color diversity also appears to be polymorphic within some of these clades [13, 14, 32]. Some of the color diversity could be explained by sexual selection, with local mate choices driving diverse color patterns in different regions [39, 40]. Likewise, wing color may be under other constraints, such as thermal tolerance or selection by predators [41]. Having robust phylogenetic hypotheses will allow for further exploration of the reasons behind this radiation in *Polythore*, where population-level analysis will be a likely next step to disentangling the complex history of these striking creatures.

## Conclusions

Our analyses suggest that the formation of the Pebas and Acre systems are associated with early diversification within the damselfly family Polythoridae. While our results indicate that the Andes were likely ancestral areas to many of the polythorid genera, the uplift of the Andes was not found to be strongly associated to tree topology, suggesting that dispersal to other regions (e.g. Amazon, Guiana Shield and Central America) played an important role in early diversification. Finally, the genus *Polythore* was the only one experiencing a significantly high rate of diversification, specifically within the Andean clade. This large-scale biogeographical analysis of a Neotropical damselfly family has yielded insights into speciation within this group and has suggested future research directions. Further exploration of the climatic and vegetation history of South and Central America in conjunction with population-level analyses may provide a better understanding of polythorid distribution than geological and geographical conditions alone.

## Methods

### Taxon sampling

A total of 48 of the 58 species of Polythoridae were included for all reconstructions presented here. All the taxa from Sanchez et al. 2018, including outgroups of other related Calopterygoid taxa (Calopterygidae, Philogangidae, Euphaeidae, and Pseudolestidae) and two non-Calopterygoidea from the Lestidae family (*Lestes temporalis* and *Lestes dryas*), are included within the analyses, with an additional 12 polythorid species new to these analyses. We included the monotypic *Chalcothore montogomery* De Marmels, 1988, 2 species of the 5 species of the genus *Chalcopteryx*, 6 of the 8 species of *Cora* s.s, 7 of the 20 species of the genus *Euthore* s.l., 3 of the 4 species of the *Miocora* genus, and 16 of the 21 species of the genus *Polythore*. Geographic origin, collector details, and Genbank Accession Numbers for all specimens are summarized in Table S6.

### DNA amplification, sequencing, and alignment

For the 12 species new to this analysis we extracted DNA from either the legs or ¼ of the pterothorax using a DNeasy Tissue Kit (QIAGEN) from each specimen following the manufacturer’s protocol. We amplified three mitochondrial and three nuclear fragments: Cytochrome Oxidase I (~ 799 bp), NADH subunit I dehydrogenase (~ 548 bp), 16S, (~ 340 bp), 28S (~ 340 bp) and 18S (~ 600 bp) (see Table S7 for a list of primers used). All gene fragments were amplified using PCR conditions as described in the associated publications for each pair of primers (Table S7) Macrogen USA Inc. laboratories (NY) performed the purification protocol for the PCR products (15 μl final volume for each primer) and the Sanger DNA sequencing. Primer contig assembly, peak chromatogram verification and the generation of per-individual consensus sequences were done using Geneious v8 [42]. All fragments were aligned using MAFFT [43] and then manually aligned in Mesquite [44]. Ribosomal genes were aligned manually with reference to secondary structure using the methods described in Kjer [45] and Kjer et al. [46]. Finally, all genes were concatenated using Mesquite for the overall analyses. We reconstructed a mid-root phylogenetic hypothesis using a Maximum Likelihood criterium in IQTree [47] including the 12 new species included within this analysis; both support values, SH-aLRT and UFBootstraps, were estimated for each node.

### Time divergence analysis

A relaxed-clock molecular dating analysis on the partitioned dataset was run using BEAST v 1.8.4 [48]. Specifically, we partitioned the gene fragments as follows: (i) We linked the sites and clock models for all mtDNA fragments and (ii) unlinked all nuclear ones from their clock and site models. We implemented the appropriate model selection for each partition: JC for 18S and 28S nDNA, we set the model to GTR + G, models obtained using the model selection tool of IQTree for ND1, 16S and COI [47]. We used lognormal relaxed clock models for all partitions, under a Yule speciation model tree prior. We estimated an unrooted topology to avoid biased node age estimation, all posterior probabilities (PP) for the nodes were estimated. Most of the fossil calibrations prior distributions were uniform considering the oldest fossil age as the maximum and the youngest fossil age as the minimum bound. For the Euphaedidae outgroup calibration we created a Lognormal distribution of all the fossil ages that accounted for all the age variation. Table 2 shows the calibrated nodes (4), stem fossils (10) and prior distributions selected for the analyses. We ran four independent analyses to ensure convergence of the MCMC; convergence was checked using Tracer 1.7 [49]. Finally, the independent runs for each treatment were combined using LogCombiner v 1.8.3 [48]. The dated ultrametric tree was obtained using TreeAnnotator v 1.8.3 [48] and visualized using Figtree v 1.4 (http://tree.bio.ed.ac.uk/software/figtree/) and the R package ggtree [50].

**Table 2:** Fossils supporting each of the node calibrations and the prior distributions selected for the Bayesian time divergence analysis. Node numbers are depicted in Fig. 1

Node/TAXA	Fossil Classification and estimated ages	Type Locality/PaleoDB	Prior distribution
Root	Odonata, Zygoptera, Eosagrionidae, *Eosagrion risi* †, Early Jurassic, Toarcian, 183–182 Ma (Handlirsch, 1920)	Germany/ Dobbertin, Mecklenburg PaleoDB 123,987	Uniform prior distribution max = **183** min = **47.8**
*Calopterygoidea*	Odonata, Zygoptera, Calopterygoidea, Calopterygidae *Sinocalopteryx shanyongensis †,* Eocene, Ypresian (56–47.8 Ma) NIGP 151367 (Lin et al., 2010) Odonata, Zygoptera, Epallagidae, *Labandeiraia europae †,* Eocene, Ypresian (56–47.8 Ma) Odonata, Zygoptera, Epallagidae, *Ejerslevia haraldi †,* Eocene, Ypresian (56–47.8 Ma) (Zessin, 2011)	Yunnan, China PaleoDB 113,892 Island of Fur, Denmark PaleoDB 123,998, 127,173 Ejerslev, Mors, Denmark PaleoDB 157,041	
Outgroup *Euphaeidae*	Odonata, Zygoptera, Epallagidae, *Labandeiraia americaborealis †,* Eocene, Bridgerian (50–46.2 Ma) 31.665A-B (Petrulevicius et al., 2007) Odonata, Zygoptera, Epallagidae, *Litheuphaea coloradensis †,* Eocene, Bridgerian (50–46.2 Ma) BMNH Pl II 562 (Petrulevicius et al., 2007) Odonata, Zygoptera, Epallagidae, *Eodichroma mirifica †,* Late/Upper Eocene, (37.2–33.9) (Cockerell 1923) Odonata, Zygoptera, Epallagidae, *Litheuphaea ludwigi †,* Eocene, Priabonian (38–33.9 Ma) (Bechly,1990) Odonata, Zygoptera, Epallagidae, *Elektroeuphaea flecki †,* Eocene, Priabonian (38–33.9 Ma) (Nel et al., 2013) Odonata, Zygoptera, Epallagidae, *Parazacallites aquisextanea †,* Oligocene, Chattian (28.1–23.03 Ma) MNHN IPM-R.06688 (Nel, 1988)	Colorado, USA PaleoDB = 107,337 Colorado, USA PaleoDB = 107,337 Texas, USA PaleoDB = 130,390 Baltic Amber, Russian Federation PaleoDB = 123,911 Baltic Amber, Poland PaleoDB = 123,215 Bouches-Du-Rhone, France PaleoDB 123,943	LogNormal; mean = 50; std. = 0.15
Ingroup/ *Polythoridae*	Odonata, Zygoptera, Polythoroidea, *Bolcathore colorata †*, Eocene, Lutetian (47.8–41.3 Ma) MCSNV I.G. 37,582 (Gentilini, 2002) Odonata, Zygoptera, Polythoroidea, *Bolcathore sp. †*, Eocene, Priabonian (38–33.9 Ma) MCSNV I.G. 37,582 (Nel and Fleck, 2014) Odonata, Zygoptera, *Protothore explicata †,* Eocene, Bartonian (41.3–38 Ma) (Cockrell, 1930)	Pesciara di Bolca, Italy PaleoDB = 122,230 Isle of Wight, United Kingdom PaleoDB = 123,960 California, USA PaleoDB = 117,537	Uniform prior distribution max = **48** min = **33.9**

### Biogeographical reconstructions

The ancestral range estimation was performed with the following software: R package Biogeobears v.0.2.1 [51] and RASP (Reconstruct Ancestral State in Phylogenies) [52]. Both software packages allowed us to customize dispersal rates matrices and time stratification events, as well as infer areas among the following historical biogeography frameworks: Dispersal-Vicariance Analysis (DIVA [53]), Statistical Dispersal-Vicariance (S-DIVA [54]), Dispersal-Extinction Cladogenesis (DEC [55]), and Bayesian inference of historical biogeography for discrete areas (BayArea [56]). In addition, Matzke [57] included a new parameter in all the models accounting for what he calls the founder-event speciation. These are described as “jumping dispersal events (J)”, which are rare events that occur when a new population colonizes a new area [57]. In particular, BioGeoBEARS has implemented model selection among six different historical biogeographic scenarios (DEC, DEC + J, DIVALIKE, DIVALIKE + J, BayArea, BayArea + J [51];). Although, Ree and Sanmartín [15] recently highlighted there are conceptual and statistical issues with the DEC + J model implemented in BioGeoBEARS, which can sometimes favor unparsimonious numbers for “jumping dispersal events”; and as a result it will not reflect a more close approximation of the “true” model of range evolution. For our analyses, we designated nine distinct geological areas based on the geological literature [1, 34, 35], which include; (A) Central America, (B) Tumbes-Choco-Magdalena, (C) North Western Andes, (D) North Eastern Andes, (E) Venezuela Highlands, (F) Central Andes, (G) Amazon Basin, (H) Guiana Shield, and (I) Brazilian Shield (Fig. 3, Table S2). The extant species distributions for each of our species at the tips of our time calibrated tree (Fig. 3) were compiled from our locality data, and specimen records information from the following collections: Florida State Arthropod Collection (FSCA), U.S National Entomological Collection (USNM), Andes Museum of Natural History (ANDES), Entomological Collection of the Universidad de Antioquia, Colombia (CEUA), and the Rutgers Newark Entomological Collection (RUN_ODO). Ranges were restricted to be comprised of at most three different areas as there are no extant species that occupy a range made up of more than three of the determined areas. Impossible adjacency range combinations were manually removed from the ranges list used by BioGeoBEARS during the inference of ancestral states. Details on the model parameters, areas allowed, dispersal probabilities and time stratification schemes for each of the all five scenarios (S0-S5) are explained in Fig. 2. All these scenarios were based on a combination of the most recent reviews concerning the Amazon Basin Formation, Miocene flooding events and Andean uplift history [1, 19, 35]. Each of these scenarios were subject to the six available historical biogeographic models of BioGeoBEARS, from the best-fit model based on the corrected Akaike Information Criterion (AICc) weights. However, following the recommendations of Ree and Sanmartín [15] we assessed model consistency by comparing among the selected BioGeoBEARS models and carefully examined the reconstructions performed for those models in both BioGeoBEARS and RASP. Afterwards, we favored the model based on best fit for the empirical information on Polythoridae and method consistency. The best selected reconstructed areas models, for each scenario were mapped over the best time calibrated phylogeny and the directionality of the dispersal and vicariance events was represented in the nodes for each major supported clade [51, 57].

### Time diversification analyses

To investigate the patterns of diversification and extinction rate variation through time and across lineages, we chose RevBayes [16, 17] to test the best diversification models and rate shifts, due to the high uncertainty associated with phylogenetic tree estimation. We calculated the Bayes Factors [58] for each pair of candidate models estimating the marginal Likelihood (mlnL) using two sampling algorithms (stepping-stone sampling [59] and path-sampling [60]) among the following models: Yule Pure-Birth Model, Birth-Death Constant Model and Episodic Birth-Death Models with multiple time intervals (4, 10 and 100 [61]). Once the best model through time was selected we calculated the speciation (λ), extinction (μ), net diversification (λ-μ) and relative extinction (λ/μ) for the best model; all plots were generated using the RevGadgets package in R [62]. In addition, we used the Branch Specific Diversification Model implemented in RevBayes [16, 17] to detect rate shifts across lineages. For all the models implemented in RevBayes [16] we assumed a uniform taxon sampling and an incomplete sampling fraction of 48/57. For the model selection, we ran a total of 5000 MCMC generations with a burnin of 1000 generations, all the parameter outputs were checked in Tracer [49] to assess the ESS, prior and posterior probability, and markov chain proper behavior. We estimated the mlnL of the models sampling the power posterior of each model for 1000 MCMC generations with a burnin of 10%. For the branch specific estimation, we assumed the heterogeneous model and we test several rate categories (*N* = 1(constant), 4 and 10) across the lineages, for each rate we obtained the marginal probabilities and Bayes Factors as explained above. For the best selected model, we wrote a file with all the estimated parameters (λ avg., μ avg., λ-μ avg., λ/μ avg. and its 95% confidence ranges) for each branch that can be mapped over our BEAST time divergence tree and was visualized using FigTree (http://tree.bio.ed.ac.uk/software/figtree/).

## Supplementary information

**Additional file 1: Fig. S1.** A. Best ML IQTree phylogenetic reconstruction for the family Polythoridae. UFBoostraps (10,000 pseudoreplicates) and SH-aLRT (1000) branch supports are above each branch. B. Geographic distribution of the family Polythoridae.

**Additional file 2: Table S2.** Most likely estimated ancestral areas for the main nodes within the Polythoridae family. The best biogeographical models for all scenarios (S0-S5), using both BioGeoBEARS (grey columns) and RASP (white columns) reconstruction methods, are shown. The designated areas include; (A) Central America, (B) Tumbes-Choco-Magdalena, (C) North Western Andes, (D) North Eastern Andes, (E) Venezuela Highlands, (F) Central Andes, (G) Amazon Basin, (H) Guiana Shield, and (I) Brazilian Shield. * indicates high uncertainty values for these nodes’ numbers correspond to Fig. 3.

**Additional file 3: Fig. S3.** Time-calibrated tree highlighting the nodes with dispersal (green), vicariance (blue) and extinction (red) events implemented in the S-DEC model for the Pebas and Acre systems (S1) scenario.

**Additional file 4: Table S4.** Multiple comparisons of the Bayes Factors and marginal likelihoods for all the diversification models tested (Yule, Birth-Death (BD), Episodic Birth-Death (EBD); the latter with multiple episodes 4, 10 or 20).

**Additional file 5: Fig. S5.** Net diversification, relative extinction, speciation and extinction rates inferred from the best diversification model (EBDN20) implemented in RevBayes.

**Additional file 6: Table S6**. Taxon Sampling, Accession numbers and Geographic location.

**Additional file 7: Table S7.** PCR conditions and Primers used in this analysis.

## Data Availability

Data are available in GenBank; see Supplementary Table S6 for accession numbers. All files for Time Divergence analyses and Biogeographical Reconstructions are published in the following repository: 10.5281/zenodo.3862741

## Abbreviations

MCRA: Most Common Recent Ancestor
RASP: Reconstruct Ancestral State in Phylogenies
ANDES: Andean uplift
ANDES O: Old Andean uplift
ANDES Y: Young Andean uplift
P&AS: Pebas and Acre Systems
P&OA: Pebas and Old Andes
P&YA: Pebas and Young Andes
