# Supplementary figures and images for: An exploration of the complex biogeographical history of the Neotropical banner-wing damselflies (Odonata: Polythoridae)
Source: BMC Evol Biol. 2020 Jun 24;20:74. doi: 10.1186/s12862-020-01638-z (PMC7315476; doi:10.1186/s12862-020-01638-z)

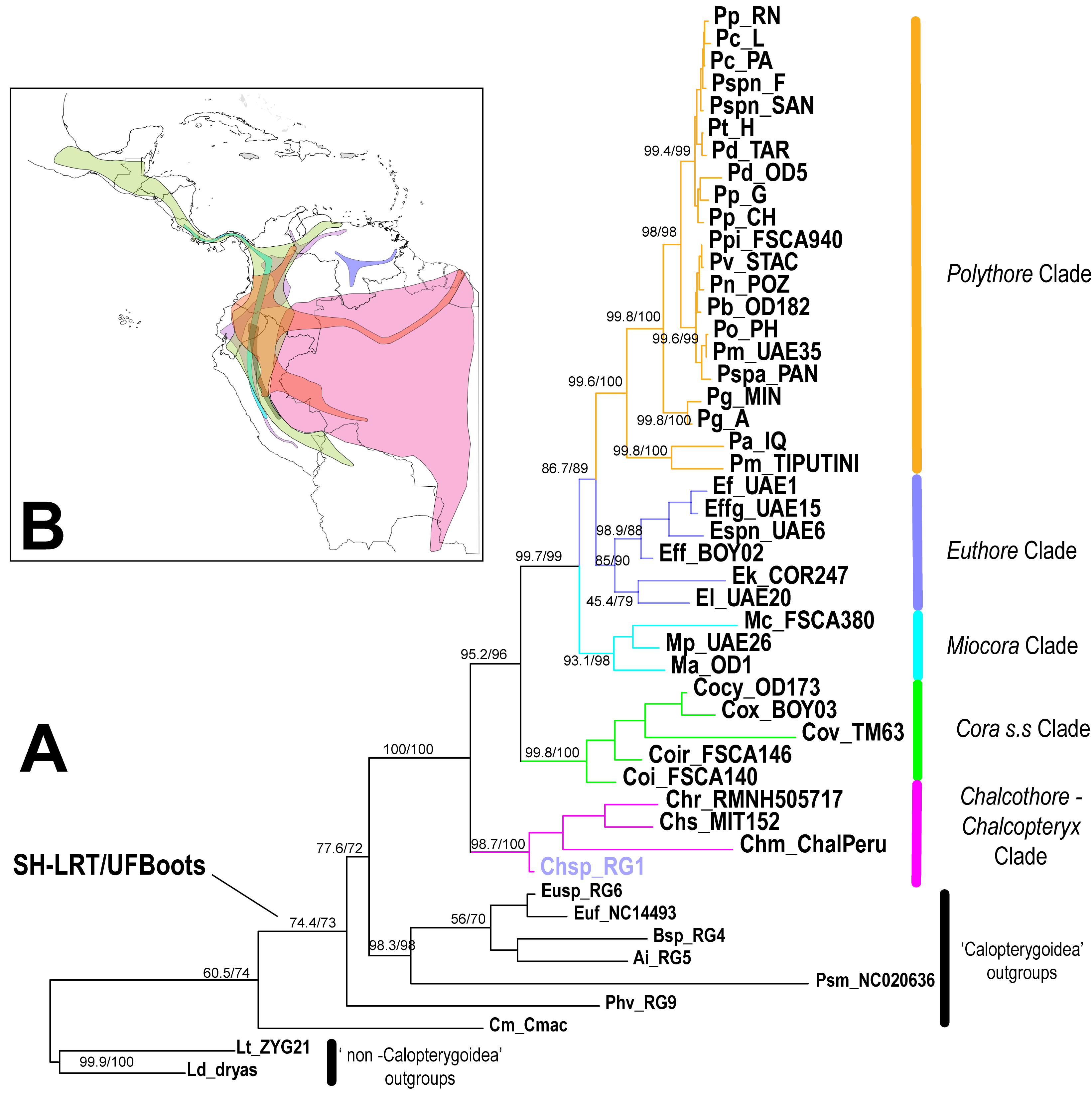

Supplement: Supplementary file 1 — Additional file 1: Fig. S1. A. Best ML IQTree phylogenetic reconstruction for the family Polythoridae. UFBoostraps (10,000 pseudoreplicates) and SH-aLRT (1000) branch supports are above each branch. B. Geographic distribution of the family Polythoridae. [file 12862_2020_1638_MOESM1_ESM.png]

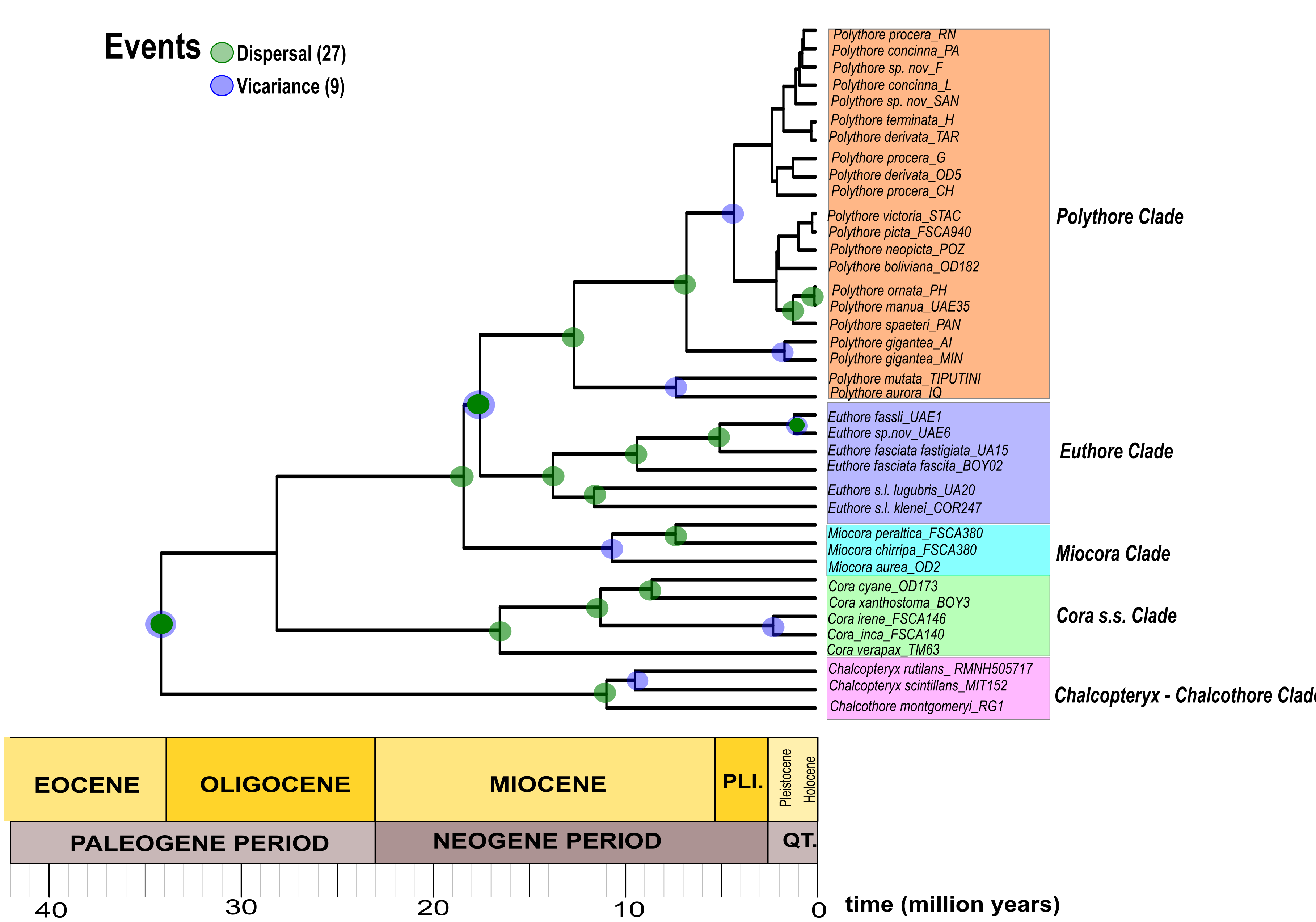

Supplement: Supplementary file 3 — Additional file 3: Fig. S3. Time-calibrated tree highlighting the nodes with dispersal (green), vicariance (blue) and extinction (red) events implemented in the S-DEC model for the Pebas and Acre systems (S1) scenario. [file 12862_2020_1638_MOESM3_ESM.png]

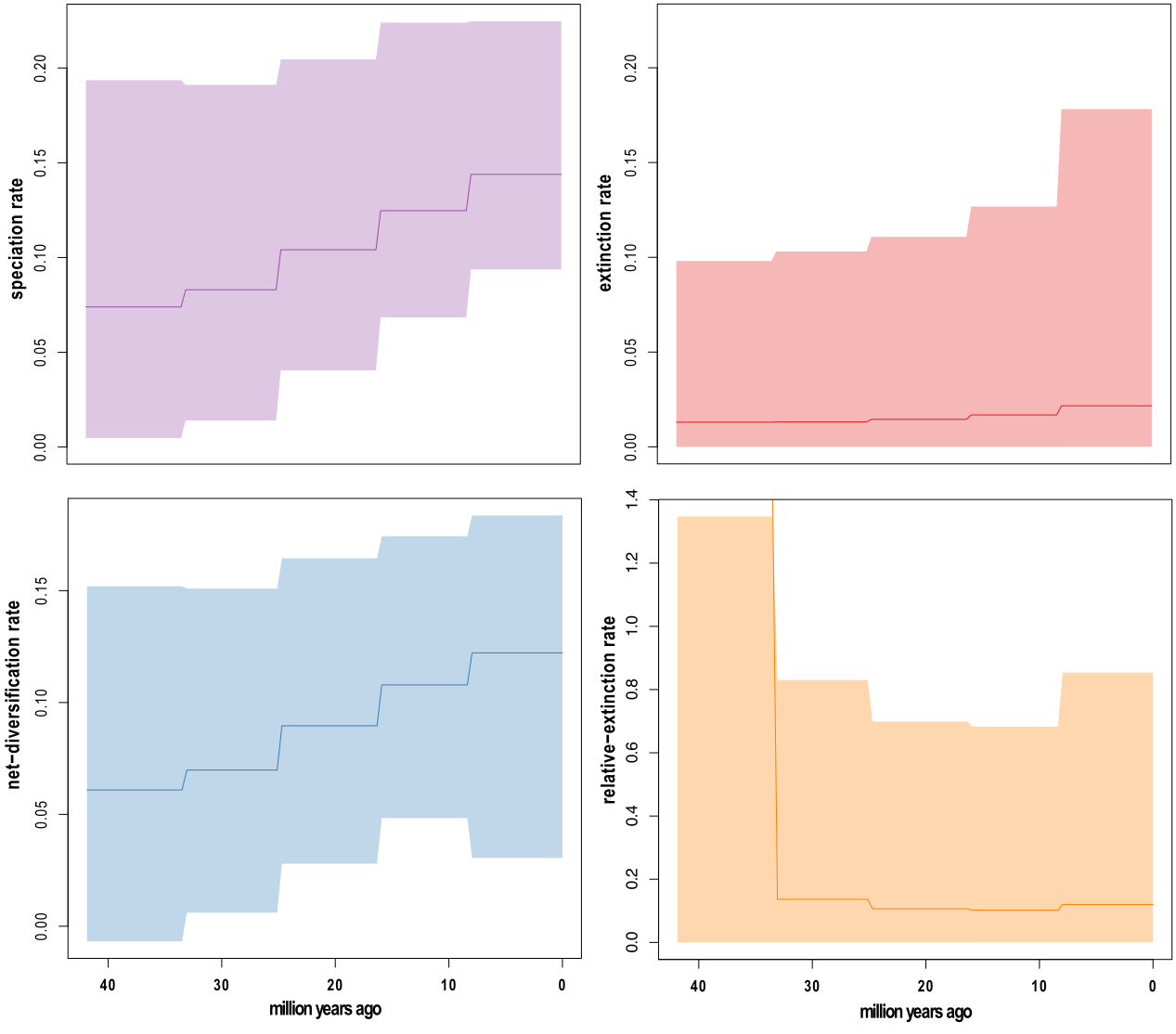

Supplement: Supplementary file 5 — Additional file 5: Fig. S5. Net diversification, relative extinction, speciation and extinction rates inferred from the best diversification model (EBDN20) implemented in RevBayes. [file 12862_2020_1638_MOESM5_ESM.png]
