# Supplementary material for: An exploration of the complex biogeographical history of the Neotropical banner-wing damselflies (Odonata: Polythoridae)
Source: BMC Evol Biol. 2020 Jun 24;20:74. doi: 10.1186/s12862-020-01638-z (PMC7315476; doi:10.1186/s12862-020-01638-z)
Supplement: Supplementary file 4 — Additional file 4: Table S4. Multiple comparisons of the Bayes Factors and marginal likelihoods for all the diversification models tested (Yule, Birth-Death (BD), Episodic Birth-Death (EBD); the latter with multiple episodes 4, 10 or 20). [file 12862_2020_1638_MOESM4_ESM.docx]

**Table S4.** Multiple comparisons of the Bayes Factors and marginal likelihoods for all the diversification models tested (Yule, Birth-Death (BD), Episodic Birth-Death (EBD); the latter with multiple episodes 4, 10 or 20).

| **Model** | **mLikelihood** | **Bayes Factors** | | | | |
| --- | --- | --- | --- | --- | --- | --- |
|  |  | Yule | BD | EBDN4 | EBDN10 | EBDN20 |
| Yule | -224 | 0 | 0 | 11 | 9 | 12 |
| BD | -221 | 0 | 0 | 140 | 110 | 153 |
| EBDN4 | -226 | 11 | 140 | 0 | 1 | 1 |
| EBDN10 | -226 | 9 | 110 | 1 | 0 | 1 |
| EBDN20 | -226 | 12 | 153 | 1 | 1 | 0 |
